# The compassionate communities connectors program: effect on healthcare usage

**DOI:** 10.1177/26323524231205323

**Published:** 2023-10-26

**Authors:** Samar M. Aoun, Natasha Bear, Bruce Rumbold

**Affiliations:** School of Medicine, Perron Institute for Neurological and Translational Science, The University of Western Australia, 8 Verdun St, Perth WA 6009, Australia; Institute of Health Research, University of Notre Dame Australia, Fremantle, WA, Australia; La Trobe University, Melbourne, VIC, Australia Perron Institute for Neurological and Translational Science, Perth, WA, Australia

**Keywords:** chronic disease, compassionate communities, connectors, cost-effectiveness analysis, end-of-life care, evaluation, healthcare usage, palliative care, social and practical needs, social connectedness, volunteers

## Abstract

**Background:**

Public health approaches to palliative and end-of-life care focus on enhancing the integration of services and providing a comprehensive approach that engages the assets of local communities. However, few studies have evaluated the relative costs and benefits of providing care using these service models.

**Objectives:**

To assess the effect on healthcare usage of a community-based palliative care program (‘Compassionate Communities Connectors’) where practical and social support was delivered by community volunteers to people living with advanced life-limiting illnesses in regional Western Australia.

**Design:**

Controlled before-and-after study/Cost-consequence analysis.

**Methods:**

A total of 43 community-based patients participated in the program during the period 2020–2022. A comparator population of 172 individuals with advanced life-limiting illnesses was randomly selected from usage data from the same set of health services.

**Results:**

Relative to controls, the intervention group had lower hospitalizations per month [Incidence rate ratio (IRR): 0.37; 95% CI: 0.18–0.77, *p* = 0.007], less hospital days per month (IRR: 0.23; 95% CI: 0.11–0.49, *p* < 0.001) and less emergency presentations (IRR: 0.56; 95% CI: 0.34–0.94, *p* = 0.028. The frequency of outpatient contacts overall was two times higher for the intervention group (IRR: 2.07; 95% CI: 1.11–3.86, *p* = 0.022), indicating the Connector program may have shifted individuals away from the hospital system and toward community-based care. Estimated net savings of $AUD 518,701 would be achieved from adopting the Connector program, assuming enrollment of 100 patients over an average 6-month participation period.

**Conclusion:**

This combined healthcare usage and economic analysis of the ‘Compassionate Communities Connectors’ program demonstrates the benefits of optimizing palliative care services using home-based and community-centered interventions, with gains for the health system through improved patient outcomes and reduced total healthcare costs (including fewer hospitalizations and readmissions). These findings, coupled with the other published results, suggest that investment in the Connectors program has the capacity to reduce net health sector expenditure while also improving outcomes for people with life-limiting illnesses.

**Trial Registration:**

Australian and New Zealand Clinical Trial Registry: ACTRN12620000326998.

## Introduction

Increased demands are being placed on palliative care services, especially with the demographic shift toward an aging population coupled with a rising prevalence of complex chronic disease.^1^ In order to maintain accessible services with a high quality of care across different settings, more sustainable models for funding and resource allocation of palliative care are required. There is evidence that the effectiveness of palliative care services can be optimized using home-based and community-centered interventions, with gains for the health system through improved patient outcomes and reduced total healthcare costs (including fewer hospitalizations and readmissions).^2^

Public health approaches to palliative and end-of-life care focus on enhancing the integration of services and providing a comprehensive approach that engages the assets of local communities. One well-documented example of community-centered palliative care is through the ‘Compassionate Communities’ model, which emphasizes the central role of community development and consumer engagement in the delivery.^3^ This approach actively involves a range of stakeholders (including those within neighborhoods, workplaces, schools, and places of worship) in facilitating caregiving networks and engaging the wider society with experiences of serious illness and dying.^4,5^ Recent evaluations of the Compassionate Communities approach indicate wide-ranging benefits, such as a population-wide reduction of hospital emergency admissions,^6^ reduced social isolation,^4^ enhanced social wellbeing,^6^ and increased community capacity and social capital to care.^4^ Abel *et al.*^6^ reported a 14% reduction in unplanned hospital admissions for participants in a complex intervention combining a compassionate communities approach and an enhanced model of primary care in the UK. Their intervention however did not rely solely on volunteers: salaried community development workers linked the referring health service with community resources.

## Description of the compassionate communities connectors program

The program reviewed in this analysis was a volunteer-led initiative designed to enhance the social networks of families living with chronic or life-limiting illnesses (the volunteers are called Connectors). This was an initiative of the South West Compassionate Communities Network in Western Australia (WA), in partnership with regional health services through the Western Australian Country Health Service (WACHS). The research trial was from July 2020 to April 2022.^7^

Palliative care, chronic disease and aged care health service teams referred families to the program, and the project coordinator matched these families with appropriate Connectors in the area. These specially trained volunteers supported existing members of the families’ social networks and enlisted the support of community members, Caring Helpers. The Connectors’ role was to mobilize existing community assets (civic organizations, informal support groups, individuals) and encourage them to contribute to the practical and social support needed by people with terminal illnesses in their community. They also offer frameworks in which partnerships can be developed to meet end-of-life needs and provide a more inclusive approach to care for people with life-limiting illnesses.^8^ Connectors attended a 2-day training course delivered by content experts. Sessions included information on public health palliative care, the importance of compassionate communities and how this project fits in, death literacy and advance care planning, grief literacy, communication skills, self-care, understanding advanced illness and the role of palliative care and chronic disease teams, and volunteer processes and procedures within the health service.

The development, implementation, and evaluation of the program have been extensively described in previous publications, demonstrating significant improvements in social connectedness, reduction in social isolation, and improved coping with daily activities.^7^ For the people with life-limiting illnesses in this program, it was found that around 80% of unmet needs were in the social domain followed by home and transport issues (17–16%), medical care (12%), and preparation for end of life (11%).^7^ Volunteering as a Connector was reported as having been a positive experience for fostering a sense of community among participants, developing relationships with other community members, and enhancing the Connectors’ emotional capacity and compassion.^8^ Families greatly appreciated the support and enablement received from Connectors. Healthcare providers were impressed with the high level of resourcefulness exhibited by Connectors and perceived a great need for the program, particularly for those socially isolated.^9^

However, it is recognized that there is an urgent need for further evidence on the effectiveness of different palliative care options.^1^ There are few studies evaluating relative costs and benefits of providing care across this range of settings and service models,^10–12^ including public health approaches such as the Compassionate Communities model.^4^ To assess the program impacts for people living with advanced life-limiting illnesses or palliative care needs in Western Australia, a healthcare usage study was conducted for the Compassionate Communities Connectors pilot program. The primary study objective was to evaluate the healthcare usage and economic impacts of this community-based palliative care program.

## Methods

### Ethics approval

As part of the establishment of the Compassionate Connectors program, ethics approval was granted by WACHS Research Ethics Committee in January 2020 for extraction of the data defined above (*WACHS HREC Program Reference RGS3419*). Apart from the referral source, hospitalization data as defined below, and basic demographic descriptors (age strata, gender and primary diagnosis), no other personal information was required for this analysis. No personal identifiers were included in any of the analyses.

### Design

A controlled before-and-after design was used to assess the effects of the intervention (enrollment in the Connectors program) for the study population relative to historical comparator (‘control’) group. This design incorporated a longitudinal component to allow assessment of both ‘before and after’ effects for the intervention group, while also accounting for background trends in health service utilization among those with late-stage illness over the follow-up period.

### Setting/Participants

In the pilot program (as described in the Introduction): (i) 20 Connectors were trained, 13 participated in the program, and 9 stayed for the whole duration of the program and were each involved on an average with three families; (ii) a total of 43 community-based patients participated and have health utilization data available, with 30 participants completing additional post-outcome measures.^7^

Participants in the Connector program were selected based on the following inclusion criteria: (i) aged 18 years and older; (ii) diagnosed with advanced cancer, chronic obstructive pulmonary disease, chronic heart failure, renal disease or other chronic conditions such as neurodegenerative conditions; (iii) record of health system usage (hospital admissions or emergency department visits) or palliative care outpatient contacts in South-West WA; (iv) identified social need.^7^ Individuals in the comparator (‘control’) population were also defined by age, residence in the South-West region of Western Australia, broad diagnostic group and record of hospital use. As will be discussed below, individuals in the control population were drawn from the same records for patient referrals and health service usage as the program participants.

### Sampling procedure

A total of 172 individuals in the comparator population were selected using stratified random sampling from the deidentified records of health system usage (hospital admissions, emergency department visits and/or outpatient contacts, as fully defined below) in South-West WA. These data sources on patient referral and health service usage were comparable to those used to select program participants. The main difference was that the time window used to select the comparator population (‘controls’) was between 2017 and 2022 inclusive, compared to 2020–2022 for the intervention group. The patient numbers in the region are not large enough to select a comparator population within the same time window as the program participants. The WACHS and other health agencies and providers assisted with the data extraction process for recorded hospitalization events in the South-West region.

Randomized control selection was conducted via a computer-generated randomization sequence using Stata SE v.17 (Texas, USA) based on age strata (that is, +/−5 years relative to participant age), gender, whether deceased status at the end of follow-up period, and primary diagnostic classification (predominantly cancer, cardiorespiratory disorders, and neurological disorders). To maximize study power and capture a broad range of clinical trajectories, four individuals in the comparator population are drawn per program participant. With 4:1 matching for 43 patients in the program, the total sample size was 215 participants (43 participants and 172 controls).

For program participants who were deceased at the time of analysis, the follow-up period for the comparator population continued to the date of death. For patients in the program who were not deceased at the time of analysis, follow-up intervals for the control group were selected from a 12- to 24-month time window preceding death.

### Measures of health service utilization

For each individual in the study population, the following data on any public hospitalization use for 12 months prior to and up to 28 months following the program commencement date were used: (i) *Emergency Department (ED) data*: date of presentation; presenting symptom; International Classification of Diseases (ICD) code; Urgency Related Group code; Triage code; if patient admitted or not; (ii) *Inpatient data*: admission date; separation date; if patient admitted from ED or by direct referral from specialist/General Practitioner (GP), etc.; ICD-10-AM code of primary diagnosis and additional diagnoses; Diagnosis Related Group; (iii) *Outpatient services*: including outpatient clinic appointments at the hospital, home visits, telephone/telehealth consultations, and multidisciplinary meetings conducted within the outpatient system.

The primary outcomes relating to health service utilization included in the final modeling were: frequency of inpatient admissions; length of hospital stay (in days); emergency department presentations; total outpatient contacts.

### Statistical analysis

For the initial stage of the analysis, simple distributional and descriptive analyses (including comparisons of means, medians and graphical displays) were used to explore program effects on frequency of inpatient admissions, length of stay, emergency department presentations, and outpatient contacts in the follow-up period.

A negative binomial mixed model was used with random effects for individuals to account for the correlation amongst repeated measures. A negative binomial model was selected to account for overdispersion in the count data. An offset was entered in the model accounting for each individual’s time in the study. The model was adjusted for age, gender, deceased status at the end of the follow-up period, and timing of the health system response to COVID-19 in Western Australia. The control group was the reference group. Incidence rate ratios (IRRs) and their corresponding 95% confidence intervals were produced.

### Economic analysis

The costs and outcomes associated with delivering the intervention *versus* the baseline were compared using cost-consequence analysis (CCA), a form of economic evaluation in which disaggregated costs and a range of outcomes are presented without generating a formal ratio or threshold (as in cost-effectiveness studies).^13^ This form of analysis is often used for evaluating interventions with multiple effects and endpoints.^13^

## Results

### Characteristics of study population

The characteristics of the study population are provided in Table 1, including demographic and clinical comparisons of the program participant group (*n* = 43) *versus* the control group (*n* = 172).

**Table 1.** Characteristics of study population.

Demographic/Clinical descriptor	Program participant group (*n* = 43)	Control group (*n* = 172)
Age in years: mean (SD)	71.3 (11.9)	72.2 (11.6)
Gender (*n*; %)	M 19 (44.2%): F 24 (55.8%)	M 76 (44.2%): F 96 (55.8%)
Primary clinical classification (*n*; %)	Cardiovascular disorder: 14 (32.6%) Cancer: 19 (44.2%) Neurological disorder: 6 (14.0%) Other: 4 (9.2%)	Cardiovascular disorder: 56 (32.6%) Cancer: 76 (44.2%) Neurological disorder: 24 (14.0%) Other: 16 (9.2%)

SD, standard deviation.

### Patterns of health service utilization

**Figure 1.**
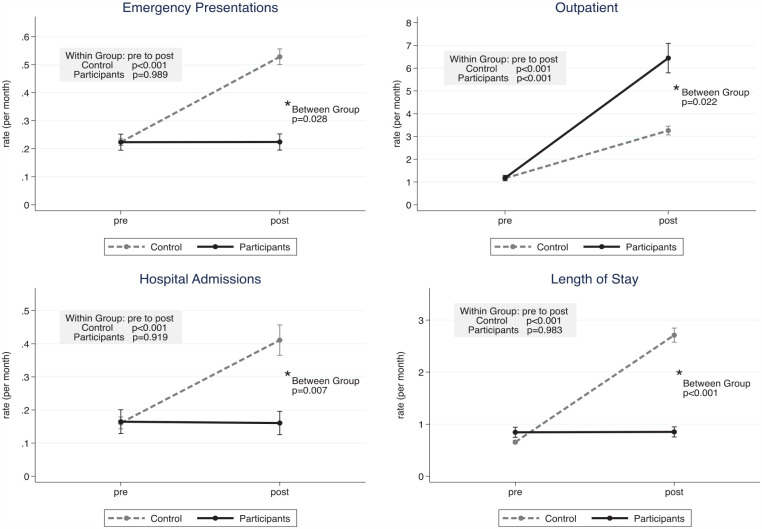
Adjusted mean rates (per month) and 95% confidence intervals from pre to post program. Showing within and between group differences, determined using negative binomial model. Adjusted for age, deceased at completion of study, timing of the health system response to COVID-19 in Western Australia and follow up period for each subject.

The average follow-up time across the study population was 25.5 months (range 12–38). The percentage deceased at the end of the follow-up period was 30.2%. Before-after differences in health service utilization events are provided in Table 2 and Figure 1.

**Table 2.** Regression outputs for health service utilization in intervention and control groups.

Outcome	Group	Rate (per month)		Within group comparison from pre to post program		Between group comparison following program	
		Pre-program	Post-program	Adjusted IRR (95% CI)^a^	*p* Value	Adjusted IRR (95% CI)	*p* Value
Frequency of hospital admissions (monthly)	Participants	0.19	0.17	0.98 (0.60, 1.58)	0.923	0.37 (0.18, 0.77)	0.007
	Control	0.20	0.42	2.56 (2.07, 3.16)	<0.001		
Inpatient length of stay (days per month)	Participants	0.73	0.76	1.01 (0.46, 2.20)	0.983	0.23 (0.11, 0.49)	<0.001
	Control	0.70	2.78	4.14 (2.87, 5.98)	<0.001		
Emergency department presentations (monthly)	Participants	0.23	0.23	1.00 (0.68, 1.47)	0.989	0.56 (0.34, 0.94)	0.028
	Control	0.24	0.55	2.36 (2.00, 2.80)	<0.001		
Outpatient contacts (monthly)^b^	Participants	1.33	7.11	5.49 (2.67, 11.29)	<0.001	2.07 (1.11, 3.86)	0.022
	Control	1.40	3.68	2.80 (2.24, 3.50)	<0.001		

a Estimates adjusted for age, gender, deceased status at the end of the follow-up period, timing of the health system response to COVID-19 in Western Australia.

b For patients deceased at the end-of-study period.

IRR, incidence rate ratio.

Baseline rates were similar across all outcomes. The control group displayed a statistically significant rise in adjusted rates from pre to post across all four outcomes (within group changes for the control group all *p* < 0.050). Overall, emergency presentations, hospital admissions and length of stay all displayed similar trends, with the control increasing their rates over time and the participant group maintaining their rates from pre to post program. This resulted in between-group difference in the post program phase for emergency presentations, hospital admissions and length of stay, with a significantly lower rates in participants compared to the control group. Emergency presentations were 44% lower, hospital admissions 63% lower and inpatient stays 77% lower in the participants versus the controls (*p* = 0.028, *p* = 0.007, *p* < 0.001 respectively).

Outpatient appointments demonstrated a different trend compared to the other three outcomes. Both the control and participants had increasing rates, but higher rates were seen post intervention in the participants, with twice the rate of outpatient appointments (adjusted between group difference IRR 2.07, 95% CI: 1.11–3.86, *p* = 0.022).

### Cost–consequence analysis

The relative costs and consequences against the control group (baseline) are summarized in Table 3. Relative costs and benefits were assessed from the perspective of the Western Australian Department of Health, assuming that this agency would be primarily responsible for operating the Connector program if this was to be implemented at a future date. Given that the program is primarily conducted in people’s homes or via phone or internet contact, there are no general fixed costs such as facility maintenance, rates, electricity and water fees incurred by the Department of Health. It is not expected that separate office space within the Department of Health would be required and this has also not been costed. Productivity changes such as time off work because of illness, treatment or caregiving were not estimated.

**Table 3.** Costs and consequences for intervention *versus* control groups for hypothetical population of 100 people with life-limiting disease over an average 6-month participation period. (All costs given in 2021/22 Australian dollars).

	Control group^a^ (standard care)	Connector program group^a^	Cost difference: intervention *versus* control group ($AUD)
**Operational and administrative costs**			
Employment of Connector program coordinators [two coordinators employed for 4 days per week for 6 months^b^]	0	92,000	**+92,000**
Regional travel for coordinators and connectors [estimated 20 km per week in rural areas for 6 months for 20 connectors and two coordinators including in-person update meetings with connectors]^c^	0	8923	**+8923**
Communication costs [standard mobile phone plan primarily for local calls for two coordinators and 20 connectors]	0	6600	**+6600**
Total			**+107,523**
**Health service utilization** ^a^			
Inpatient length of stay	2,154,345	1,368,189	**−786,156**
Emergency department presentations	422,013	178,988	**−243,025**
Outpatient contacts	412,475	815,432	**+402,957**
Total			**−626,224**
Net savings			−518,701

a Assuming care of 100 patients with life-limiting disease over an average 6-month participation period.

b Based on current (2023) salary rate for Program Coordinators in WA Department of Health, assuming one coordinator is responsible for 10 connectors, who in turn manage 50 patients in the program (i.e. on average five participants per connector) over an average 6-month participation period.

c 78 cents per kilometer (based on Australian Tax Office estimates for 2022–2023).

The principal costs relate to the operation and administration of the Connector program, as well as ongoing regional travel and communication costs for coordinators and connectors. As noted above, it is assumed that most interactions between coordinators and connectors will occur *via* mobile phone or internet. To provide an example of the predicted impact of the intervention, based on the pilot study, it is estimated that one coordinator employed 4 days per week will be responsible for on average 10 connectors at one time, with each connector in turn responsible for an average of five individuals with life-limiting disease over an average 6-month participation period. In the basic model below, costs are given assuming that two coordinators have been employed, overseeing 20 connectors who would in turn be responsible for 100 individuals with life-limiting illness.

Baseline costs per hospital length of stay were estimated by multiplying costs per admission day at the main regional hospital ($2679) and then multiplying by the average monthly number of admission days for the control group (on average 1.34 days per month). For emergency department utilization, baseline costs were estimated by multiplying costs per contact for most common type of triage code at the main regional hospital ($1332) and then multiplying by the average monthly number of ED contacts for the control group (on average 0.52 ED contacts per month). Baseline costs per outpatient contacts were estimated by multiplying costs for most common appointment type at the main regional hospital ($211) and then multiplying by the average monthly number of events for the control group (on average 3.25 outpatient contacts per month for patients deceased at the end of the study period). Baseline costs were then compared to the adjusted effects associated with the intervention on hospital length of stay, emergency department presentations and outpatient contacts, respectively.

All costs are given in 2021/22 Australian dollars.

## Discussion

This health care usage analysis for the Compassionate Connectors pilot program indicated a significant shift in health utilization patterns for the Connector (‘intervention’) group *versus* a comparator population. Relative to controls, the intervention group had lower hospitalizations per month, less hospital days per month and less emergency department admissions. In the controls, we saw a rise in health care usage over time across all outcomes. We did not see a reduction in health care usage in participants, but instead they maintained their rates for hospitalizations days per month and emergency presentations. Relative to the control group, this pattern resulted in a 63% less hospital admissions, 77% less days in hospital and 44% less emergency presentations. However, a different pattern was observed for outpatient usage, with a higher frequency observed in the participants resulting in rate of outpatient contacts overall being two times higher for the intervention group.

The cost–consequence analysis provided an indication of the relative economic impacts of the Connector (‘intervention’) program. Compared to the costs and benefits associated with baseline care in the comparator population, the net saving associated with implementing the Connector program was estimated at $AUD 518,701, assuming enrollment of 100 people with life-limiting disease and follow-up over an average 6-month participation period.

There are a number of possible explanations for the observed pattern of the effectiveness study results, in which fewer hospital and ED admissions and shorter lengths of stay were coupled with a higher number of outpatient contacts on average. Based on direct observations of the patients during the pilot study, it was apparent that Connectors and caring helpers were assisting with transportation to health services and even with making medical appointments on behalf of the patients. This additional support would tend to encourage patients to attend their outpatient appointments in a timely manner rather than delaying or missing them, thus increasing the number of these visits for the intervention group. It is also probable that patients in the program spent less time in hospital because they could rely on Connectors and caring helpers outside the hospital to provide assistance and emotional support.^8^ It was noted by the study coordinators that many connectors made contact with patients ‘after-hours’. As a result, at least for some patients, the community networks may be providing a feasible alternative to prolonged hospital care or more frequent admissions. Therefore, the fact that the number of inpatient days for program participants are lower suggests that these patients may be more likely to return home, possibly because of the more supportive environment or community-based options for better management, as opposed to being admitted and remaining in the hospital system.

Overall, the findings of this study provide support for the Connector model of care in addressing unmet patient and family end-of-life needs. As noted in the Introduction, the reported benefits of the Compassionate Communities approach include reduced social isolation,^4^ enhanced social wellbeing,^6^ and increased community capacity.^4^ For these participants, the majority of support was sourced from the outer informal networks or the community at large (or called ‘externally facilitated networks’) rather than from within the inner informal networks of participating families, reflecting that half of the patients referred to the program were living alone.^7,9^ The Connector study provided particular benefits for people who live alone or who are socially isolated in more rural communities that are out of the frequent reach of formal services.^7^ In other circumstances, and for other client groups, Connectors assisted with linkages to a wider range of services, such as encouraging chronic disease services to realize their potential as providers of generalist palliative care.^8^

More broadly, as Cardona-Morrell *et al.* have shown, both non-beneficial treatment in hospital at the end of life^14^ and inappropriate hospital use for older people near the end of life^15^ are significant costs to health systems. Their systematic reviews of the problems suggest that approximately 35% of patients near the end of life receive non-beneficial treatments, which can include tests, futile treatments, and Cardiopulmonary Resuscitation (CPR). They also found a wide range across different health systems in the proportion of elderly patients retained in hospital for essentially social reasons, because community alternatives were lacking. Overall, they found that inappropriate admissions, and inability to discharge inpatients no longer requiring hospital care, arose from a range of system factors, social and family factors. They pointed to the need for community alternatives to manage end-of-life care, skilling of staff to manage terminal illness in non-acute settings, and the need to continue to monitor clinically inappropriate admissions and inappropriate length of stay. Clearly the Connectors program addresses both these concerns.

Groeneveld *et al.*^1^ have identified a range of important attributes of palliative care systems to ensure that service delivery is both effective and acceptable. These include supporting funding models that encourage early access to palliative care where appropriate, that provide services in the most appropriate location, that minimize the financial burden on patients and families, and that provide a system that can be readily navigated without excessive administration and transaction costs.^1^ Swerissen and Duckett^16^ have also argued that when a range of high-quality end-of-life services are available to them, people with end-stage conditions are much more likely to die at home and are less likely to be admitted to hospital. Many of these goals would also potentially be achieved by supporting a Connector model of care based in regional communities.

In relation to other health initiatives, the results of the Connector study also support recent approaches to regional care currently being implemented by the WACHS. The health service concept of Place-Based Care is that all hospital and community services occur within an area or ‘place’, such as a country town, which will tend to have strong person-centered culture where health services are delivered collaboratively across service and professional boundaries. For example, where possible, many people with end-stage illness prefer to die comfortably at home or in a home-like environment with minimal pain and suffering, surrounded by friends and family and the care services they need.^16^ The WA Country Health Service deemed the Connectors program to be well aligned with their Place-Based Care strategy and was adopted by the service as standard practice.

### Limitations

Potential limitations in the effectiveness analyses included the risk of unrecognized factors amongst patients in the program and controls that differentially affects health utilization pathways. These could include lack of comparability in terms of socioeconomic status and degree of geographical isolation. Given the deidentified nature of the control data and privacy concerns, it was also not possible for the research team to contact individuals directly to gather additional personal or clinical information. The living alone status could not be controlled for as the health system ICD code related to this variable is only assigned if relevant to the reason of hospital admission and only for an inpatient admission. Therefore, someone could still be living alone, but this status would not be put in as an ICD code.

The study focused on hospital usage and did not include the costs of primary care such as GP or nursing or allied health time. For a minority of individuals in the control group (30 people or 17.4% of total controls), the comparisons of health service utilization included pre-COVID-19 follow-up periods. In general terms, the Western Australian population and health system were not as significantly disrupted by COVID-19 as many other regions, in part because the state implemented strict border closures from March 2020 to March 2022 which in large part shielded the entire population. However, some reorganization of health service access did occur in response to the pandemic, and an indicator variable reflecting recruitment during the pandemic period was introduced into the model.

The long survival period of the intervention patients was due to the upstream recruitment from the chronic disease care service, not just from the palliative care service: nearly half of those who were recruited from palliative care were too close to death and many died soon after being enrolled in the program, mainly because they were referred too late to the palliative care service.^7^

### Implications

This partnership involved specialist palliative care, generalist palliative care as provided by chronic disease services, the civic organizations and compassionate communities working together to support the families, which is the optimal practice model promoted as the New Essentials in Palliative Care.^17^ To effect and maintain a system change, all the four cogs of the new essentials model must operate in synchronization to create an effective, affordable and sustainable end-of-life care system. Health departments have a role to play in a public health model because they act as the linkage chain between the four cogs: They should encourage services to start conversations and allocate resources to develop relevant partnerships identified through these conversations; and they need to get into community development as well as direct service provision and prepare the way by developing partnerships with civic and community networks.

Fundamental to the Connector program, and to all public health strategies arising from a compassionate communities approach, is the need to establish a ‘shared care’ model that can link the contributions of health services, community resources, and the informal networks of care that surround individual patients. This raises issues of privacy and accountability that are managed somewhat differently in different jurisdictions. We have noted in a previous article^18^ the constraints imposed on volunteers by the risk management strategies required by health services.

A further implication is that clinicians who see the need for a public health approach need to initiate this as informed citizens in their own local communities, not as professionals expanding the health service in which they are employed. Improving death literacy and grief literacy, training programs in network mapping and network enhancement and on how to use public resources for the benefit of people at the end of life are a must for all service providers, not just for the community. Various models for community initiation of a compassionate communities approach are available^19,20^ in addition to our earlier description of the setting up of the Compassionate Connectors Program.^7–9^

## Conclusion

To our knowledge, this is the first study to have evaluated the healthcare usage and associated savings of adopting the ‘Compassionate Communities Connectors’ approach in palliative care. The analyses indicate the potential benefits of uptake of public health approaches to managing life-limiting disease, with a greater emphasis on partnerships between health services, civic institutions and communities. As noted by McCaffrey *et al.*,^10^ palliative care requires ‘a broad range of services provided by diverse disciplines across all health care sectors’. Meeting social needs reduces demand on clinical services. The economic analysis demonstrates the benefits of optimizing palliative care services using home-based and community-centered interventions, with gains for the health system through improved patient outcomes and reduced total healthcare costs (including fewer hospitalizations and readmissions). These quantitative findings, coupled with the other published qualitative results from this study, suggest that investment in the Connectors program has the capacity to shift patterns of service utilization away from its disproportionate reliance on inpatient care, thereby reducing net health sector expenditure while also improving outcomes for people with life-limiting illnesses.

## Declarations

**Ethics approval and consent to participate:** The study was conducted according to the guidelines of the Declaration of Helsinki and approved by the WACHS Human Research Ethics Committee (RGS3419). Participants provided written informed consent prior to the start of data collection.

**Consent for publication:** Not applicable.
